# Bilateral Synchronization of Hippocampal Early Sharp Waves in Neonatal Rats

**DOI:** 10.3389/fncel.2019.00029

**Published:** 2019-02-07

**Authors:** Guzel Valeeva, Azat Nasretdinov, Veronika Rychkova, Roustem Khazipov

**Affiliations:** ^1^Laboratory of Neurobiology, Kazan Federal University, Kazan, Russia; ^2^Aix-Marseille University, INMED, Institut National de la Santé et de la Recherche Médicale (INSERM), Marseille, France

**Keywords:** hippocampus, neonate, sharp waves, bilateral, synchronization

## Abstract

In the neonatal rodent hippocampus, the first and predominant pattern of correlated neuronal network activity is early sharp waves (eSPWs). Whether and how eSPWs are organized bilaterally remains unknown. Here, using simultaneous silicone probe recordings from the left and right hippocampus in neonatal rats *in vivo* we found that eSPWs are highly synchronized bilaterally with nearly zero time lag between the two sides. The amplitudes of eSPWs in the left and right hippocampi were also highly correlated. eSPWs also supported bilateral synchronization of multiple unit activity (MUA). We suggest that bilateral correlated activity supported by synchronized eSPWs participates in the formation of bilateral connections in the hippocampal system.

## Introduction

Interhemispheric synchronization is a hallmark of neuronal network activity in the hippocampal system. Bilateral recordings revealed high level of synchronization of various hippocampal activity patterns including theta and gamma oscillations, sharp waves (SPWs) and paroxysmal activity patterns (Suzuki and Smith, 1987; Buzsáki, 1989, 2015; Buzsáki et al., 2003; Carr et al., 2012; Shinohara et al., 2013; Wang et al., 2014; Pfeiffer and Foster, 2015; Benito et al., 2016; Tanaka et al., 2017; Villalobos et al., 2017). Interhippocampal synchronization is supported by several mechanisms including commissural connections between hippocampi and synchronous input from entorhinal cortex (Mizuseki et al., 2009; Shinohara et al., 2012; Benito et al., 2016; Fernández-Ruiz et al., 2017). However, when and how bilateral synchronization emerges in the hippocampal system remains unknown.

During development, neuronal networks generate particular patterns of correlated activity that participate in the formation of neuronal circuits (Katz and Shatz, 1996; Khazipov and Luhmann, 2006; Blankenship and Feller, 2010; Hanganu-Opatz, 2010; Colonnese and Khazipov, 2012; Luhmann and Khazipov, 2018). Early activity patterns are expressed in a way of local (patchy) intermittent activity bursts such as spontaneous movement driven spindle- and gamma-bursts in somatosensory cortex (Khazipov et al., 2004; Yang et al., 2009, 2013; Mohns and Blumberg, 2010; Minlebaev et al., 2011; Akhmetshina et al., 2016) or retina/cochlea driven waves in visual and auditory cortex, respectively (Hanganu et al., 2006, 2007; Colonnese and Khazipov, 2010; Ackman et al., 2012; Babola et al., 2018). These early forms of neocortical activity occur largely asynchronously in the two hemispheres, however. Lateralization is particularly prominent in somatosensory cortex (Yang et al., 2009; Marcano-Reik et al., 2010). It is also present in visual system where only a small proportion of events, presumably generated by retinal waves originating from binocular retina regions is bilaterally synchronized (Hanganu et al., 2006; Ackman et al., 2012), and in the auditory system where cochlea driven activities are biased towards predominant contralateral cochlear input (Babola et al., 2018).

In the neonatal hippocampus, the first and predominant pattern of correlated neuronal network activity is early SPWs (eSPWs; Leinekugel et al., 2002; Karlsson et al., 2006; Mohns et al., 2007; Mohns and Blumberg, 2008; Marguet et al., 2015; Valeeva et al., 2019). eSPWs are intermittent events characterized by sharp negative potentials in CA1 strata lacunosum-moleculare (*sl-m*) and radiatum (*sr*) reversing at pyramidal cell layer (*pcl*), and synchronous neuronal firing in CA3, CA1 and dentate gyrus. eSPWs are generated by cooperation of inputs from medial entorhinal cortex and intrinsic intrahippocampal connections. eSPWs are typically triggered by spontaneous myoclonic neonatal movements and are preceded by activity bursts in superficial layers 2–3 of medial entorhinal cortex (Karlsson et al., 2006; Marguet et al., 2015; Valeeva et al., 2019). However, whether eSPWs support bilateral synchronization of activity in the left and right hippocampus as adult SPWs do (Suzuki and Smith, 1987; Buzsáki, 1989, 2015) remains an open question.

Here, we addressed this question by bilateral recordings from the right and left CA1 hippocampus using silicone probes in neonatal P5-7 non-anesthetized head restrained rat pups. We found that nearly all eSPWs occur in both hippocampi synchronously with almost zero time lag and that eSPWs support high level of bilateral synchronization of neuronal activity.

## Materials and Methods

### Ethical Approval

This work has been carried out in accordance with EU Directive 2010/63/EU for animal experiments and all animal-use protocols were approved by the French National Institute of Health and Medical Research (INSERM, protocol N007.08.01) and Kazan Federal University on the use of laboratory animals (ethical approval by the Institutional Animal Care and Use Committee of Kazan State Medical University N9-2013).

### Animal Preparation

Wistar rats of either sex from postnatal days (P) 5–7 were used. Preparation of the animals for recordings was performed under deep isoflurane anesthesia the day before recording as previously described (Akhmetshina et al., 2016; Valeeva et al., 2019). Briefly, while under isoflurane anesthesia the skull was cleared of skin and periosteum and covered by dental cement, leaving ≈5 mm^2^ windows above the left and right hippocampi. The wound was treated with xylocaine (2%) and chlorhexidine (0.05%). Animals were warmed up and returned to the litter to recover from surgery and did not receive additional medications during recordings.

### Electrophysiological Recordings

Recordings were performed from head-restrained non-anesthetized rats. A metal ring was fixed to the skull with dental cement and *via* ball-joint to a magnetic stand. Animals were surrounded by a cotton nest and heated *via* a thermal pad (35–37°C). During recordings, animals were regularly fed with heated milk and continuously monitored for any sign of pain or discomfort, and if such occurred, the animals were sacrificed with an overdose of urethane.

Extracellular recordings of local field potentials (LFPs) and multiple unit activity (MUA) were performed along the CA1—dentate gyrus axis of the dorsal hippocampus using a pair of 16-site linear silicon probes with 50 μm separation distance between the electrodes (four animals) or two eight-shank 64-site probes with 200 μm separation distance (two animals; NeuroNexus, Ann Arbor, MI, USA). Two craniotomies of 0.2–0.3 mm diameter each were performed above the left and right hippocampi. DiI coated electrodes were placed using stereotaxic coordinates (Khazipov et al., 2015). A chloride silver wire, placed in the neocortex, served as a ground electrode. Signals from extracellular recordings were amplified and filtered (10,000×; 0.15–10 kHz) using DigitalLynxSX amplifier (Neuralynx, Bozeman, MT, USA) and digitized at 32 kHz. From 30 min to an hour of spontaneous activity were recorded in each animal.

### Histology

After recordings the animals were deeply anesthetized with urethane (3 g/kg, intraperitoneally) and perfused intracardially with 4% paraformaldehyde and 1% glutaraldehyde (Sigma). The brains were removed and left for fixation for a few days. One-hundred micron-thick coronal slices were cut using a Vibratome (Thermo Fisher Scientific, Waltham, MA, USA). Electrode positions were identified from the DiI tracks overlaid on the microphotographs of sections after cresyl violet staining.

### Data Analysis

Wideband recordings were preprocessed using custom-written functions in MATLAB (MathWorks, Natick, MA, USA). eSPWs were detected semi-automatically from down-sampled (1,000 Hz), bandpass filtered (3–100 Hz, Chebyshev type 2 Filter) LFP signal. All events reaching the amplitude greater than 1.5 standard deviations at filtered LFP on *sl-m* and *pcl* channels (negative and positive peaks, respectively) were first considered as putative eSPWs. To discard movement and static artifacts, LFP segments from −1 s to 1 around the eSPW were visually inspected. The eSPW onset was defined as a time when the first LFP derivative in *sl-m* reached a threshold of 2 mV/s. Raw data were filtered using 250–4,000 Hz bandpass wavelet filter (Daubechies 4) and spikes were detected as negative events exceeding −3.5 standard deviations of filtered signal. Time lags between the left and right hippocampus were calculated from peri-onset time histograms smoothed by moving average filter (4 ms window for eSPWs onsets and 20 ms for MUA). *Z-scores* were estimated on the basis of a shuffled artificial data as described previously (Valeeva et al., 2019).

### Statistics

Statistical analysis was performed using the MATLAB Statistics toolbox. Group comparisons were done using one- and paired-sample Wilcoxon signed-rank tests. *P*-value of less than 0.05 was considered significant. Correlations between variables were estimated using the Pearson (*r*) correlation coefficients. Unless indicated, data are presented as mean ± SD.

## Results

**Figure 1:**
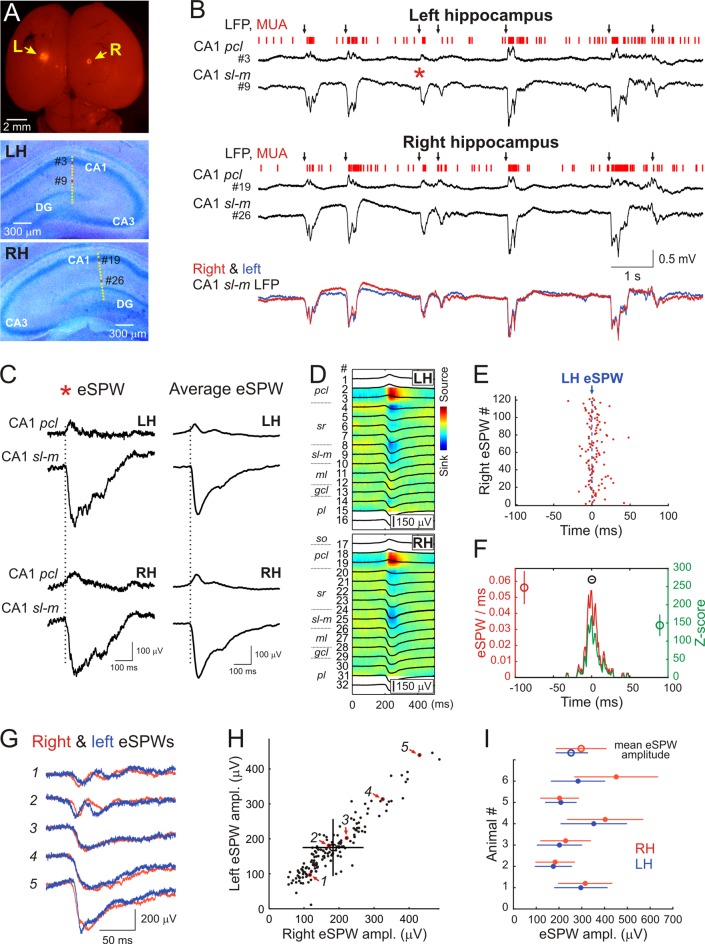
Interhemispheric synchronization of hippocampal early sharp waves (eSPWs) in neonatal rats. **(A)** *Top*: the microphotograph of DiI traces at insertion sites of two silicone probes on the surface of the left (L) and right (R) brain hemispheres in a P6 rat pup. *Middle—Bottom*: recording sites of 16-channel probes overlaid on cresyl violet stained coronal slices of the left (LH, middle) and right (RH, bottom) hippocampi. (**B)** Simultaneous local field potential (LFP) and multiple unit activity (MUA) recordings from the left hippocampus and right hippocampi from CA1 pyramidal cell layer (*pcl*, recording sites #3 and #19 on panel **A**) and strata lacunosum-moleculare (*sl-m*, recording sites #9 and #26 on panel **A**). Black arrows above the traces indicate eSPWs. Bottom, overlaid *sl-m* LFP traces from left (blue) and right (red) hippocampus. **(C)** The eSPW from panel (**B**; red asterisk) and average left eSPW-triggered LFP in the left and right hippocampus on expanded time scale. **(D)** Left eSPWs-triggered LFPs (black) in the left and right hippocampi overlaid on CSD maps. **(E)** Left eSPW onset-triggered raster plot of right eSPW onsets. **(F)** Left eSPW onset-triggered normalized PETH of right eSPW onsets (red) and corresponding *z-score* values (green). Group averages (mean ± SD; *n* = 6 animals) show the peak value of normalized PETH (red circle), the peak value of *z-score* (green circle) and the time lag between left and right eSPWs (black circle). **(G)** Five example traces of eSPWs recorded simultaneously in CA1 *sl-m* of left (blue) and right (red) hippocampi. **(H)** Relationships between left and right eSPW amplitudes recorded in CA1 *sl-m* layer (animal #2). Average amplitude values are indicated by an open circle with error bars corresponding to SD. Red arrows indicate the data points (outlined with red) corresponding to eSPWs shown on panel **(G)**. See also Supplementary Figure S1 for all animals. **(I)** Amplitude averages of eSPWs in the left (blue) and right (red) hippocampi of six P5–7 rats (closed circles) and group values (open circles). Error bars show SD.

In the present study we performed simultaneous LFP and MUA recordings from the dorsal part of the left and right CA1 hippocampus in six non-anesthetized head-restrained postnatal days [P] 5–7 rats (Figure 1). The location of the recording sites was identified during *post hoc* analysis of the DiI electrode tracks in coronal sections (Figure 1A).

**Figure 2:**
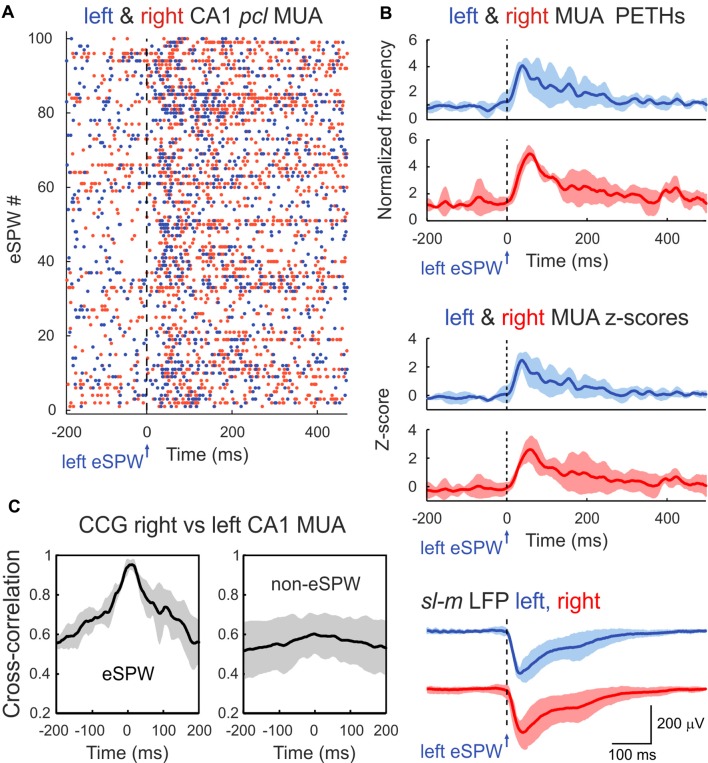
Bilateral synchronization of CA1 MUA during eSPWs. **(A)** Left eSPW onset-triggered raster plot of CA1 *pcl* MUA in the left (blue dots) and right (red dots) hippocampus. **(B)** Left eSPW onset-triggered and normalized to the baseline MUA peri-event time histograms (PETHs) in left and right hippocampi (top), corresponding MUA *z*-scores (middle) and average LFP (bottom). **(C)** Normalized cross-correlograms of MUA in right vs. left hippocampus within the time window of eSPWs (left) and during non-eSPW epochs (right). Cross-correlograms were normalized to the maximum value during eSPW epochs. **(B,C)** Group averages from three animals. Shaded areas show confidence intervals. MUA PETHs, *z-scores* and cross-correlograms were smoothed using a 20 ms-long sliding window.

In keeping with previous results, hippocampal activity on both sides was characterized by the ripple-lacking eSPWs which were associated with a negative sharp potential below the CA1 *sr* and *sl-m* and polarity reversal at the *pcl*, and often followed by “tails” (Leinekugel et al., 2002; Karlsson et al., 2006; Mohns et al., 2007; Mohns and Blumberg, 2008; Marguet et al., 2015; Valeeva et al., 2019; Figures 1B–D). eSPWs attained maximal negativity in *sl-m* (left: 253 ± 68 μV; right: 297 ± 111 μV; *n* = 6) and their current-source density profile was characterized by the two main sinks, one in *sr* and another in *sl-m* (Figure 1D) as reported previously (Valeeva et al., 2019). eSPWs were also associated with MUA bursts (Figures 1B, 2A–C). The frequencies of eSPWs in the left and right hippocampus were of 3.8 ± 1.5/min and 3.9 ± 1.6/min, respectively (*p* = 0.44). As shown in Figure 1B, eSPWs in the right and left hippocampus were also highly synchronized. We further assessed the bilateral co-occurrence probability and the time lags between eSPWs in the right and left hippocampi. We found that 97 ± 4% of eSPWs in the right hippocampus co-occurred with eSPWs in the left hippocampus (*z*-score = 144 ± 30) and, vice versa, 96 ± 4% of eSPWs in the left hippocampus co-occurred with eSPWs in the right hippocampus (*z*-score = 140 ± 29) within a ±100 ms time window (Figures 1E,F). The vast majority of eSPWs occurred simultaneously with nearly zero time lag between hemispheres, although some eSPWs occurred with a time lag in the range of up to 10 ms. On average, the time lags separating eSPWs in the left and right hippocampi were of 0.3 ± 2.3 ms. These results indicate that eSPWs are highly synchronized bilaterally.

eSPWs in the left and right hippocampi also highly correlated in amplitude. While the eSPWs’ amplitude varied in both sides, larger in amplitude eSPWs in one hippocampus were associated with larger eSPWs in the contralateral hippocampus (Figures 1G–I, Pearson’s *r* = 0.84 ± 0.14; *n* = 6; *p* < 0.001; see also Supplementary Figure S1 for all animals). Also, eSPWs were slightly more ample in the right hippocampus (*p* = 0.03).

In agreement with the results from previous studies, eSPWs were associated with an increase in MUA in CA1 *pcl*. MUA peri-event time histograms (PETHs) triggered by left eSPW onsets attained maximal values of 0.04 ± 0.01 spikes/ms (4.2 ± 0.2-fold increase above baseline; *z-score* 2.6 ± 0.5) and 0.05 ± 0.02 spikes/ms (5.0 ± 0.4-fold increase above baseline; *z-score* 2.6 ± 0.8) with time lags of 41 ± 8 ms and 60 ± 4 ms (*p* > 0.05) in the left and right hippocampi, respectively (Figures 2A,B; *n* = 3 rats; animals with MUA frequency <5/s were excluded from MUA analysis). Co-occurrence of eSPWs in the left and right hippocampi supported bilateral MUA synchronization during eSPWs that was evident on the MUA cross-correlograms within a time window of ±1 s from the eSPW onset. Peak of MUA cross-correlation during eSPWs attained 0.027 ± 0.014 and showed a time lag of 9 ± 9 ms between the left and right hippocampi. Bilateral MUA cross-correlation was also observed during the non-eSPW epochs, but its level was lower (0.017 ± 0.008 with a time lag of 18 ± 9 ms) than during eSPWs (Figure 2C).

## Discussion

Our main finding is that eSPWs are highly synchronized and support correlated neuronal activity in the left and right hippocampus in the rat pups *in vivo*. Previously, several forms of synchronized bilateral activity in the developing hippocampal system have been described *in vitro*. For example, giant depolarizing potentials (GDPs), recurrent neuronal network discharges originating in CA3 network are synchronized bilaterally in the preparation of interconnected hippocampi *in vitro* (Khalilov et al., 1997; Leinekugel et al., 1998). Epileptiform discharges induced by various epileptogenic agents also propagate between hippocampi and bilaterally synchronize hippocampal activity in this preparation (Khalilov et al., 1997, 1999, 2005; Khazipov et al., 1999). Lesion of the ventral hippocampal commissure or pharmacological suppression of commissural action potential propagation results in complete bilateral desynchronization of GDPs and paroxysmal activities indicating that commissural communication is pivotal for their bilateral synchronization (Khalilov et al., 1999; Khazipov et al., 1999). Consistent with these findings* in vitro*, we found perfect bilateral synchrony in eSPWs in neonatal rat pups *in vivo*. Bilateral eSPWs synchronization likely involves CA3-CA3 commissural connections as in the case of GDPs and paroxysmal events* in vitro* as described above. This is supported by activation of CA3 neurons during eSPWs also manifested by a current sink of eSPWs in CA1 *sr*, where CA3-CA1 synapses are located (Valeeva et al., 2019). However, bilateral eSPWs synchronization *in vivo* may also involve synchronous bilateral inputs from entorhinal cortex that is evidenced by synchronous eSPWs’ current sinks in CA1 *sl-m*, where synapses from the entorhinal cortex are located (*ibid*). This suggests that the activity bursts in superficial layers of entorhinal cortex preceding hippocampal eSPWs are also bilaterally synchronized. In the future, it would be of interest to determine mechanisms involved in bilateral activation of entorhinal cortex in relation to eSPWs and spontaneous body movements, which reliably trigger entorhinal-hippocampal activity in neonatal rats (Karlsson et al., 2006; Marguet et al., 2015; Valeeva et al., 2019).

In conclusion, we have shown that bilateral synchronization of activity in the hippocampal system emerges early during postnatal development and that early bilateral synchronization is supported by highly correlated eSPWs. Bilateral synchronization of eSPWs likely contributes to the development of synaptic connections between hippocampi by means of synchronization of neuronal activity and activity-dependent plasticity. Our results also provide further evidence that eSPWs are a developmental prototype of adult SPWs, which also display a high level of bilateral synchrony (Suzuki and Smith, 1987; Buzsáki, 1989, 2015).

## Data Availability

Original and processed data, and signal processing and analysis routines are available on request from the authors.

## Author Contributions

RK and GV conceived the project. GV and VR performed the experiments. AN, GV and VR analyzed the data. RK wrote the article.

## Conflict of Interest Statement

The authors declare that the research was conducted in the absence of any commercial or financial relationships that could be construed as a potential conflict of interest.

## Supplementary Material

FIGURE S1 Relationships between left and right eSPW amplitudes recorded in CA1 *sl-m* of 6 animals. Pearson’s *r* values and corresponding *p*-values are shown above the plots.
