# Supplementary figures and images for: Bilateral Synchronization of Hippocampal Early Sharp Waves in Neonatal Rats
Source: Front Cell Neurosci. 2019 Feb 7;13:29. doi: 10.3389/fncel.2019.00029 (PMC6374346; doi:10.3389/fncel.2019.00029)

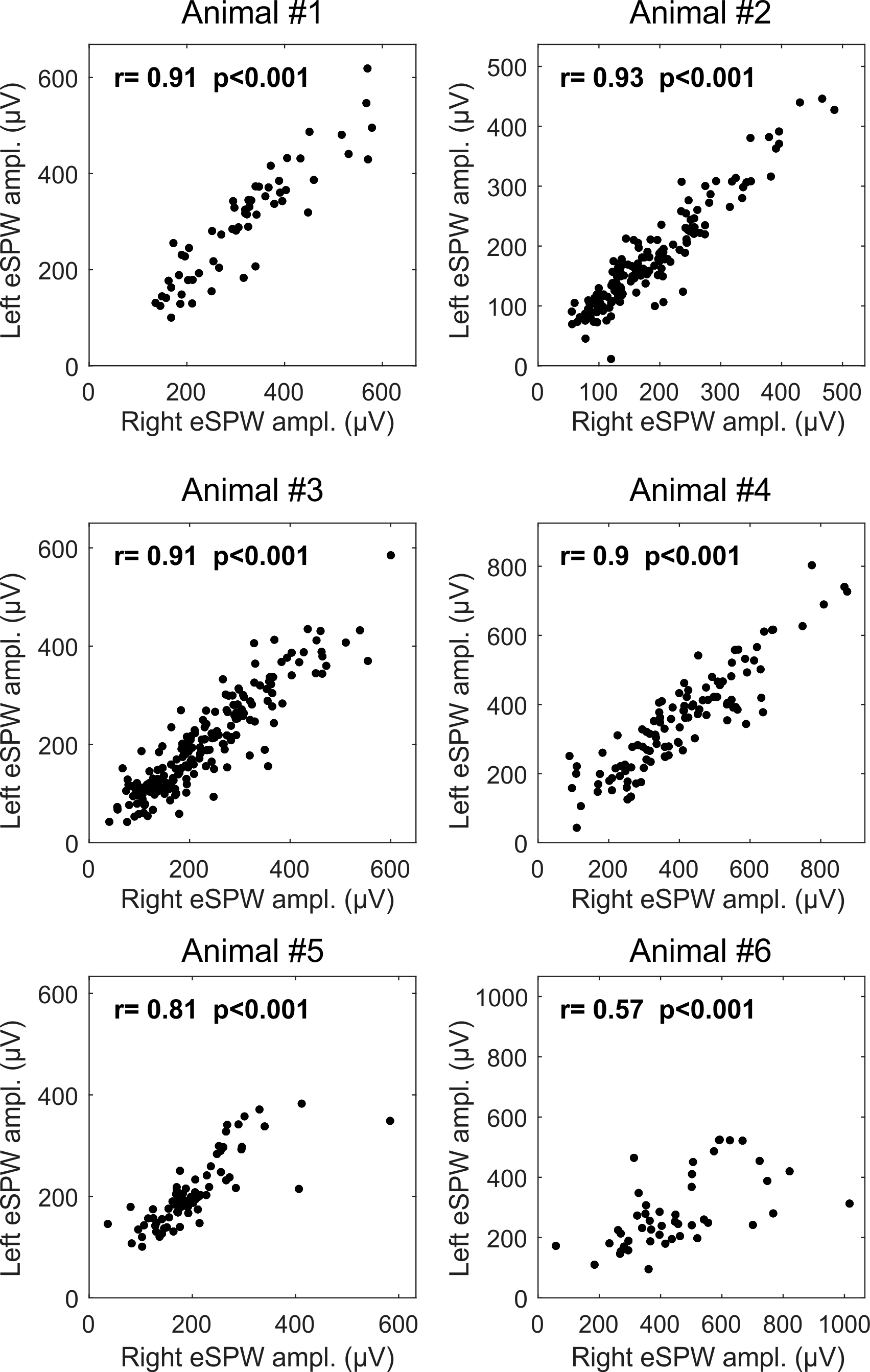

Supplement: FIGURE S1 — Relationships between left and right eSPW amplitudes recorded in CA1 sl-m of 6 animals. Pearson’s r values and corresponding p-values are shown above the plots. [file Image_1.TIF]
